# The effect of body posture on cognitive performance: a question of sleep quality

**DOI:** 10.3389/fnhum.2014.00171

**Published:** 2014-03-27

**Authors:** Markus Muehlhan, Michael Marxen, Julia Landsiedel, Hagen Malberg, Sebastian Zaunseder

**Affiliations:** ^1^Department of Psychology, Institute of Clinical Psychology and Psychotherapy, Technische Universität DresdenDresden, Germany; ^2^Department of Psychology, Neuroimaging Center, Technische Universität DresdenDresden, Germany; ^3^Section of Systems Neuroscience, Department of Psychiatry and Psychotherapy, Technische Universität DresdenDresden, Germany; ^4^Department of Electrical and Computer Engineering, Institute of Biomedical Engineering, Technische Universität DresdenDresden, Germany

**Keywords:** heart rate variability, N-back, alertness, arousal, sleep quality, posture, fMRI, ECG

## Abstract

Nearly all functional magnetic resonance imaging (fMRI) studies are conducted in the supine body posture, which has been discussed as a potential confounder of such examinations. The literature suggests that cognitive functions, such as problem solving or perception, differ between supine and upright postures. However, the effect of posture on many cognitive functions is still unknown. Therefore, the aim of the present study was to investigate the effects of body posture (supine vs. sitting) on one of the most frequently used paradigms in the cognitive sciences: the N-back working memory paradigm. Twenty-two subjects were investigated in a randomized within-subject design. Subjects performed the N-back task on two consecutive days in either the supine or the upright posture. Subjective sleep quality and chronic stress were recorded as covariates. Furthermore, changes in mood dimensions and heart rate variability (HRV) were assessed during the experiment. Results indicate that the quality of sleep strongly affects reaction times when subjects performed a working memory task in a supine posture. These effects, however, could not be observed in the sitting position. The findings can be explained by HRV parameters that indicated differences in autonomic regulation in the upright vs. the supine posture. The finding is of particular relevance for fMRI group comparisons when group differences in sleep quality cannot be ruled out.

## INTRODUCTION

Neuroimaging investigations such as functional magnetic resonance imaging (fMRI) take place in an environment that is much different from a standard laboratory setting. Several factors like the confined space, scanner noise, or the uncontrollability of the situation have been shown to elicit anxiety and stress (e.g., MacKenzie et al., 1995), which in turn can affect behavioral and neural data (Muehlhan et al., 2013). Beside these environmental factors, the most important difference between a standard laboratory setting and neuroimaging investigations is body posture. Whereas non-imaging investigations of cognitive processes are usually performed sitting upright in front of a monitor, subjects are lying in supine position during fMRI scanning. This essential difference has been discussed to affect physiological and psychological processes and renders comparisons of measurements inside and outside the scanner difficult (Raz et al., 2005; Duncan and Northoff, 2013).

On the physiological level, evidence suggests that the difference in orthostatic load between sitting and supine posture leads to changes in firing rate of baroreceptors (Rau and Elbert, 2001; Duschek et al., 2013). It has been suggested that a decrease in baroreceptor firing in the upright posture contributes to elevated arousal, as evident in increased EEG beta activity (Cole, 1989). In turn the supine posture has been associated with attenuated levels of arousal and has been discussed as a sleep promoting factor. Furthermore, Perini and Veicsteinas (2003) demonstrated that several components of the heart rate variability (HRV) spectrum differ between sitting and lying. During the supine position the high frequency component (HF: 0.15–0.40 Hz), an indicator for parasympathetic activity, showed a higher amplitude compared to the upright posture. During sitting, however, the low frequency component (LF: 0.04–0.15 Hz) was sharper and higher compared to supine posture. The LF component includes both parasympathetic and sympathetic modulation, but can be seen as an indicator for sympathetic activity at least when expressed in normalized units (Camm et al., 1996). Thus, the supine position is characterized by higher parasympathetic activity and less sympathetic activity compared to the upright position. The different levels of autonomic activation also contribute to differences in mental fatigue and sleepiness between postures (Caldwell et al., 2000; Caldwell et al., 2003). In general, evidence suggests that subjects become more fatigued (Krauchi et al., 1997; Sharafkhaneh and Hirshkowitz, 2003; Romeijn et al., 2012) and fall asleep earlier (Cole, 1989) when lying compared to an upright posture. The posture based changes summarized above can also interact with cognitive and affective processes (Duschek et al., 2013). Reviewing the literature on body posture and cognitive performance, some early studies found evidence that more hallucinatory reports are produced (Morgan and Bakan, 1965) and that more autobiographical information can be remembered (Berdach and Bakan, 1967) in a supine compared to a sitting position. A more recent investigation has shown that the supine posture improves target detection in visual extinction patients (Peru and Morgant, 2006). The latter finding, however, could not be observed in healthy controls showing a better performance during sitting. Other studies demonstrated that the performance of several tasks such as problem solving (Schulman and Shontz, 1971), resolving anagrams (Lipnicki and Byrne, 2005), and detection of auditory stimuli and peri-threshold odors (Lundstrom et al., 2006, 2008) improve in an upright compared to the supine position. Moreover, higher anger evocation (Harmon-Jones and Peterson, 2009) and higher anticipatory anxiety (Lipnicki and Byrne, 2008) have been observed during an upright sitting or standing compared to a supine posture. Thus, it seems that the majority of studies observed a beneficial effect of an upright posture on cognitive performance. However, the effect of posture on a large body of cognitive functions is still poorly understood.

One of the most frequently used paradigms in cognitive, clinical, and pharmacological neuroscience is the N-back working memory task, in which participants need to identify whether the number in the current trial is identical to the number N trials earlier or not. Therefore subjects performed alternating 1-back and 2-back blocks on two consecutive days in either a supine or a sitting position for a duration of 18 min on each day. To investigate physiological differences between postures, electrocardiograms (ECG) were recorded and HRV measures were assessed. Subjective mood (alertness, calmness, and valence) was recorded over the course of the experiment. Based on the above literature findings, we hypothesized that: (1) Subjects show poorer N-back task performance in the supine compared to the sitting posture, (2) HRV parameters show higher levels of normalized parasympathetic HRV activity in the supine posture and, consequently, higher levels of normalized sympathetic activity in the upright position, and (3) Subjects get more fatigued in the supine compared to the sitting position. Because the quality of sleep and chronic stress have been shown to contribute to mental fatigue particularly in the supine posture (Akerstedt et al., 2004), sleep quality and chronic stress levels were investigated as potentially interacting variables.

## MATERIALS AND METHODS

### PARTICIPANTS AND PROCEDURE

**FIGURE 1:**
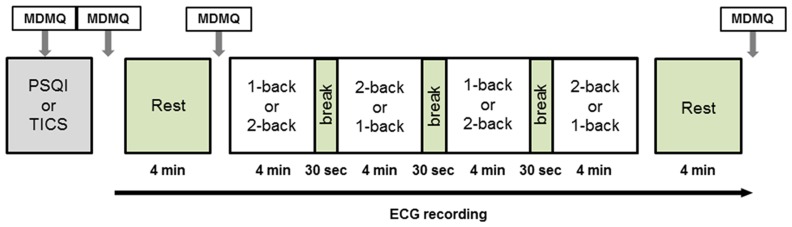
**Study protocol.** Electrocardiography was recorded continuously from the beginning of the initial rest period to the end of the final rest period. The protocol was identical on both days of examination with the exception of posture. MDMQ, Multidimensional Mood Questionnaire; PSQI, Pittsburgh Sleep Quality Index; TICS, Trier Inventory for the Assessment of Chronic Stress.

Twenty-five healthy volunteers (female *n* = 12, mean age: 21.6 ± 2.2; male: *n* = 13, mean age: 26.8 ± 2.41) were recruited via flyers displayed on the Technische Universität Dresden campus. Exclusion criteria were any cardiovascular, neuroendocrine, or psychiatric disease. Participants received course credit when requested. All participants gave their written informed consent. The study was approved by the Ethics Committee of the Technische Universität Dresden (EK: 281092012). Participants arrived either at 11 am or at 1 pm at the Institute of Biomedical Engineering and were tested on two consecutive days in either a lying or a sitting posture. The time of measurement was kept constant over both days for the same participant. The order of postures (lying vs. sitting) was counterbalanced between days. After entering the lab, the participant was informed about the study protocol and filled out three questionnaires (please see below). Then a short training phase of the cognitive task (N-back paradigm) followed. After the training, adhesive electrodes were fixed on the participant’s chest, and the participant was placed on a chair sitting upright or on an examination table in a supine position. A 4-min resting ECG measurement followed before the cognitive task (18 min) began. Finally, a second resting ECG measurement of 4 min was recorded. Participants were instructed not to close their eyes during the rest periods. Several mood assessments were conducted during the course of the experiment using the multidimensional mood questionnaire (MDMQ). Please see **Figure 1** for an illustration of the study protocol. Three participants were excluded from further analysis due to poor quality of the ECG data and one participant was excluded from behavioral analyses due to extremely high error rate.

### TASK

An N-back paradigm was used to assess working memory performance. The workload was modulated by alternating 1-back and 2-back conditions. Each condition was presented twice in blocks of 4 min each (see **Figure 1**). Whether the paradigm starts with a 1-back or a 2-back block was counterbalanced between subjects. In each block, series of white numbers reaching from zero to nine were presented centrally on a black background. Subjects had to indicate whether the current number was the same as the one in the previous trial (1-back) or the one two trials ago (2-back). Responses were given by right index finger button presses for “yes” and right middle finger button presses for “no”. Subjects were instructed to respond as fast and accurate as possible. The numbers were surrounded by a white rectangular frame. Immediately after the response was given, a feedback was displayed by a change of the frame color to green (correct) or red (incorrect). Per block, 120 numbers were shown. Each trial consisted of a 1-s stimulation period and a 1-s inter-trial interval. The response period was 2 s. The display was presented via a video projector either on the ceiling in case of the supine condition or on the wall in case of the sitting position.

### QUESTIONNAIRES

On the first examination day, the Pittsburgh Sleep Quality Index (PSQI; Buysse et al., 1989) was filled out by the participants before the actual experiment started. The PSQI is a self-rated questionnaire to assess sleep-quality in a four-week time interval. The 19 items of the PSQI include measures of (a) sleep quality, (b) sleep latency, (c) sleep duration, (d) habitual sleep efficiency, (e) sleep disturbances, (f) use of sleeping medication, and (g) daytime dysfunction. A total score was calculated from all items and used for further analyses. On the second day of examination the Trier Inventory for Assessment of Chronic Stress (TICS) was used to measure chronic stress. The TICS yields nine scales with a total of 57 Items to assess stressful experiences within the last three month. The scales are (a) work load, (b) social stress, (c) pressure to succeed, (d) work discontent, (e) excessive demands, (f) lack of social acceptance, (g) social strain, (h) social isolation, and (i) chronic worries. Twelve of the 57 Items were used to build a screening scale for chronic stress experience, which was used for further analyses (Schulz and Schlotz, 2002; Schlotz, 2010). To measure short term fluctuations in mood dimensions during different parts of the experiment, the German version of the MDMQ (Steyer et al., 1994, 1997; for the English version please see: http://www.metheval.uni-jena.de/mdbf.php) was filled out four times: (1) at the beginning of the experiment (“baseline measurement”), (2) after the task training phase and electrode attachment (to control for potential changes due to the experimental setting itself), (3) after the first rest period, immediately before the cognitive task, and (4) immediately after the experiment (to assess differences before and after the task performance). The MDMQ measures the three dimensions alertness (awake-tired), calmness (clam-nervous), and valence of mood (good mood-bad mood) by a four-point Likert scale and is suitable to measure changes in mood dimensions within several minutes. The MDMQ could be subdivided in two parallel short forms (A and B) with 12 items each. The two parallel forms were used in alternating order.

### PHYSIOLOGICAL MEASUREMENTS

During the experiment, a single-channel ECG, a plethysmogram, and thoracic respiratory movements were recorded. Measurements were amplified and digitized using ADInstruments PowerLab 16/35 together with ADInstruments FE132 biosignal amplifier. ECG was acquired by adhesive electrodes (Liquid Gel, Vivomed) attached to the upper part of the body (modified limb lead II, Rautaharju et al., 1980). A plethysmogram was taken from the earlobe by using a reflective plethysmograph (MLT1060EC, ADInstruments). Respiration was recorded from the chest by a piezoelectric respiratory belt (MLT1132, ADInstruments). All recordings used a sampling rate of 1000 Hz. After recording, data was exported from the recording software (LabChart v7.3.5, ADInstruments) to Matlab (The MathWorks, Inc.) for further analysis.

### DATA ANALYSES

#### Behavioral data

Median reaction times (RTs) and accuracy rates were calculated for every subject, work load (1-back/2-back) and posture (supine/upright). We expected the median to provide more power than the mean for our within-subject design (Whelan, 2008). RTs shorter than 100 ms were counted as errors and were not considered for further analysis. This cut-off is common practice in cognitive experiments because it has been demonstrated that processes like the stimulus perception and the initiation of a motor response require a minimum of 100 ms (Whelan, 2008). RTs longer than 1900 ms were excluded because these RTs may be related to inattentive processes instead of the process of interest. Statistical analyses were conducted using SPSS 21. The means of median RTs as well as the accuracy rates were analyzed using 2 × 2 analyses of variances (ANOVAs) for repeated measurements with the within-subject factors work load and posture. In a second step, we used analyses of covariance (ANCOVAs) to test for the covariates sleep quality (PSQI total score) and chronic stress (TICS screening scale). Greenhouse–Geisser adjustments were used when appropriated. Subsequently, Pearson correlations of RTs and accuracies with PSQI total score and TICS screening scale were calculated. Additionally a 2 × 2 ANOVA for repeated measurements with the factors examination day and work load was performed to control for potential learning effects.

The three mood ratings were analyzed using 2 × 4 ANOVAs for repeated measurements with the within-subject factors posture (supine/upright) and assessment point (1–4). Sleep quality (PSQI total score) and chronic stress (TICS screening scale) were included as covariate. Greenhouse–Geisser adjustments were used when appropriated.

#### Physiological parameters

As far as it concerns the physiological measurements, only the heart rate was considered for further analysis. Heart rate was extracted from the ECG by applying an automated QRS detector (modified version of Pan-Tompkins algorithm, Pan and Tompkins, 1985). After automated detection, erroneous QRS detections were corrected manually and the series of beat-to-beat-intervals (BBIs) was derived. To account for outliers introduced by arrhythmic events and abnormal variability in the instantaneous heart rate, the BBIs were filtered by an adaptive filter (Wessel et al., 2007). The filtered BBIs served as basis for the further calculation of HRV parameters. Our analysis focused on selected parameters according to the Task Force of the European Society of Cardiology and the North American Society of Pacing and Electrophysiology (Camm et al., 1996). The two time domain parameters SDNN (SD of all NN intervals) and RMSSD (square root of the mean squared difference between adjacent NN intervals) were calculated. The total power (TP, defined as the power in the frequency range ≤0.4 Hz), low (LFnu: 0.04–0.15 Hz) and high frequency (HFnu: 0.15–0.4 Hz) components normalized with respect to TP, as well as the LF/HF ratio were calculated for further analyses in the frequency domain parameters (see **Table 1** for an overview). All HRV parameters were extracted from 4-min intervals (at rest or during each N-back block). In order to derive parameters in the frequency domain, the filtered BBIs were first resampled to 4 Hz using the method proposed by Berger et al. (1986). Subsequently, an estimate of the power spectral density (PSD) was obtained based on an autoregressive model of 15th order. Calculations were done in Matlab. For selected tasks, routines provided by the Biosig Toolbox for Matlab (Vidaurre et al., 2011) were incorporated. The resulting parameter values were analyzed using ANOVAs. At first, 2 × 2 repeated measures ANOVAs with the factors posture and work load were used to calculate main effects and interactions between posture and work load. Second, 2 × 4 repeated measures ANOVAs with the factors posture and time period was used to calculate main effects and interactions of changes over time and between postures. Because 1-back and 2-back tasks were presented in alternating order and were randomized over subjects and days to avoid order effects, the HRV parameters from the first two blocks of the task (e.g., 1-back and 2-back) and the last two blocks of the task were (e.g., 2-back and 1-back) averaged. This approach results in the four time periods Rest 1, Block 1, Block 2, and Rest 2 (see **Figure 1**). Greenhouse–Geisser adjustments were used where appropriated.

**Table 1:** Selected time- and frequency-domain measures of HRV.

Variable	Units	Description: time-domain measures
SDNN	ms	Standard deviation of all NN intervals
RMSSD	ms	The square root of the mean squared difference between adjacent NN intervals

**Variable**	**Units**	**Description: frequency-domain measures**

TP	ms^2^	Variance of all NN intervals over the temporal segment
LF/HF		Ratio LF [ms^2^]/HF [ms^2^]
LFnu	n.u.	LF power in normalized units LF/(TP-VLF) × 100
HFnu	n.u.	HF power in normalized units HF/(TP-VLF) × 100

*According to the Task Force of The European Society of Cardiology and The North American Society of Pacing and Electrophysiology (Camm etal., 1996). TP, total power; LF, low frequency; HF, high frequency; ms, miliseconds; n.u., normalized units.*

## RESULTS

### MOOD

**FIGURE 2:**
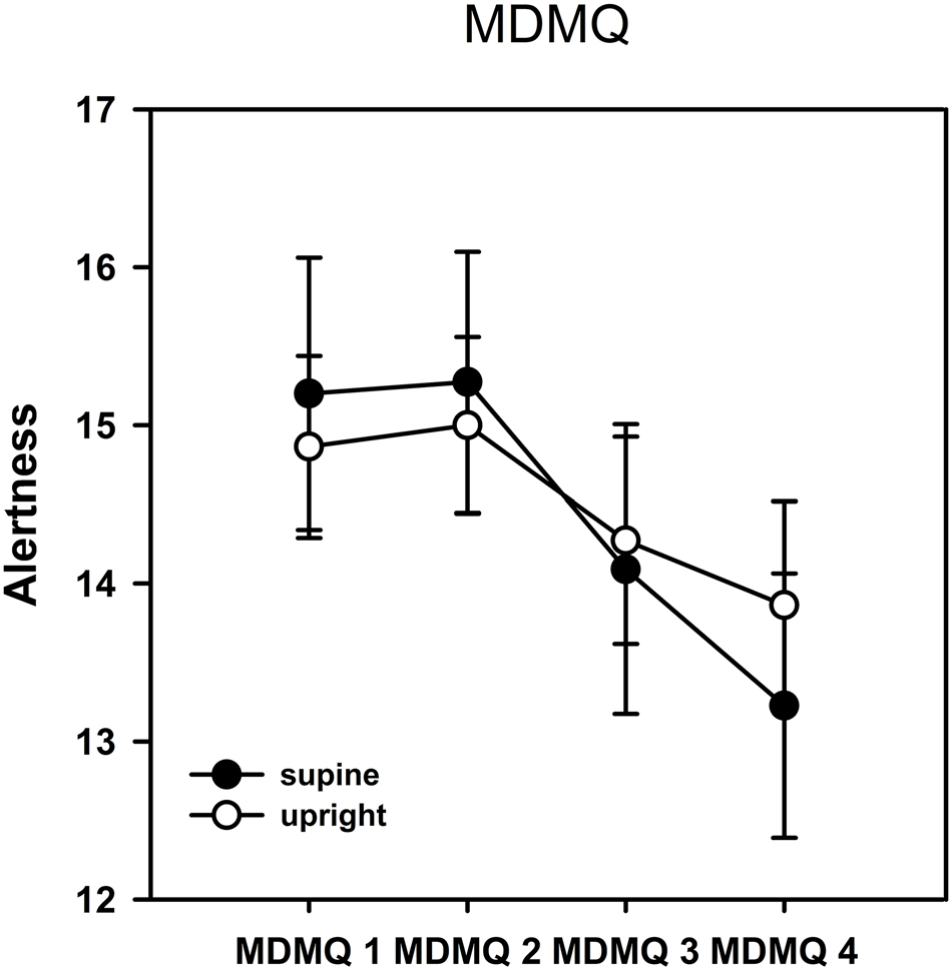
**Results from the Multidimensional Mood Questionnaire (MDMQ)–alertness scale.** Error bars indicate SEM. Alertness decreased significantly over time but there was no significant difference between postures. No significant time or posture effects were observed on the calmness or valence scales.

As described in the methods section, mood was analyzed in the three dimensions alertness, calmness, and valence. The ANOVA of the dimension alertness yielded a significant main effect of the factor time [*F*(3,19) = 5.930, *p* = 0.001, η^2^ = 0.238] indicating a change of alertness in the time course of the experiment. It is obvious from **Figure 2** that alertness decreased significantly over time. No main effect of posture and no interaction between posture and time were observed. We also could not find any significant effects on the dimensions calmness and valence.

### BEHAVIORAL DATA

#### Reaction times

**FIGURE 3:**
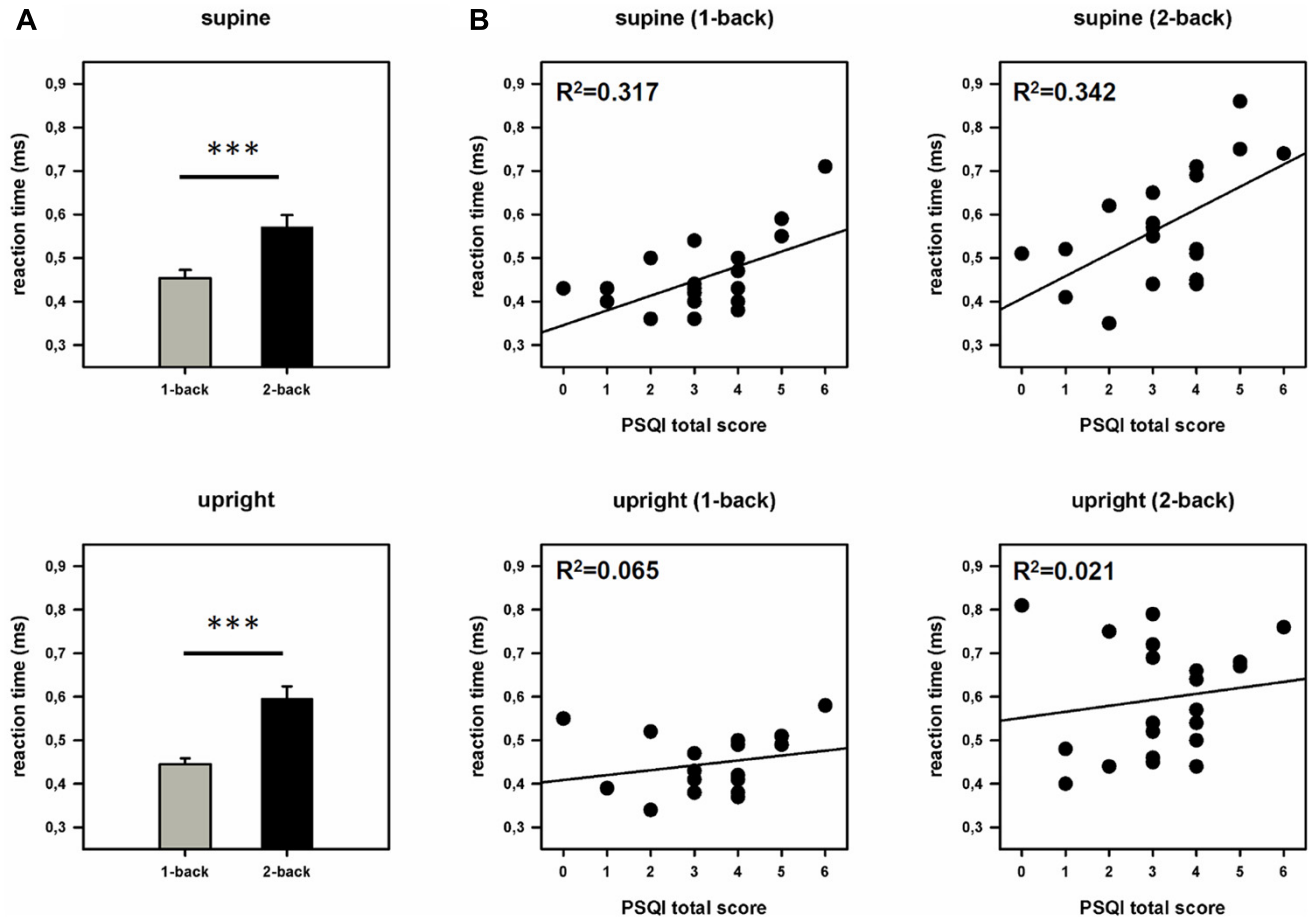
**Behavioral data. (A)** Reaction times (RTs) during both work load conditions and postures. Error bars indicate SEM. **(B)** Scatter plots and coefficients of determination (*R*^2^). Displayed is the relationship between sleep quality (PSQI total score) and reaction times (RTs) in the supine and the upright posture. ***p < 0.001.

The ANOVA (work load × posture) revealed a significant main effect of the factor work load [*F*(1,20) = 51.329, *p* = 0.001, η^2^ = 0.720], but no main effect of posture or significant interaction between posture and work load. In addition we performed the analysis with the factor “examination day” as covariate. This analysis also yielded only a significant main effect of the factor work load [*F*(1,19) = 51.302, *p* < 0.001, η^2^ = 0.730]. A main effect of the factor posture or interactions between posture and work load could not be found. Subsequent pairwise comparisons showed that subjects react faster in the 1-back (449.25 ms) compared to the 2-back task (583.65 ms) but do not differ significantly between the supine (512.59 ms) and the upright posture (520.31 ms). Interestingly, *after* integrating sleep quality as a covariate, the ANCOVA revealed a significant main effect of the factor work load [*F*(1,19) = 4.730, *p* = 0.042, η^2^ = 0.199], and of the factor posture [*F*(1,19) = 5.782, *p* = 0.027, η^2^ = 0.233]. Moreover, we found a significant effect of the covariate (PSQI total score) [*F*(1,19) = 6.004, *p* = 0.024, η^2^ = 0.240] and a significant interaction between the posture (supine/upright) and the covariate (PSQI total score) [*F*(1,19) = 5.926, *p* = 0.025, η^2^ = 0.238] but no interaction between work load and posture. Subsequent correlational analysis revealed significant positive correlations between RTs and PSQI total score in the supine (1-back: *r* = 0.563, *p* = 0.008; 2-back: *r* = 0.585, *p* = 0.005) but not in the upright position (1-back: *r* = 0.255, *p* = 0.264; 2-back: *r* = 0.146, *p* = 0.527; see **Figure 3**). Note that a high PSQI total score indicates poor sleep quality. In other words, the results show that the poorer the sleep quality the longer the RTs but only in the supine position. No significant effect of the covariate could be found after integrating the TICS screening scale instead of the PSQI. In the ANOVA (examination day × work load), we also observed main effects of the factors examination day [*F*(1,20) = 30.950, *p* < 0.001, η^2^ = 0.607] and work load [*F*(1,20) = 50.521, *p* < 0.001, η^2^ = 0.716] as well as a significant interaction between these factors [*F*(1,20) = 20.165, *p* < 0.001, η^2^ = 0.502]. Subsequent pairwise comparisons indicate that participants responded significantly faster on the second than on the first day of examination at both levels of workloads (1-back: day 1: 463.33 ms, day 2: 435.24 ms, *p* = 0.016; 2-back: day 1: 637.62 ms, day 2: 529.05 ms, *p* < 0.001).

#### Accuracy

Analysis of variance (work load × posture) yielded a significant main effect of the factor work load [*F*(1,20) = 11.656, *p* = 0.003, η^2^ = 0.368]. No significant main effect of the factor posture or significant interactions between posture and workload could be found. Analysis with the “examination day” as potential confounder revealed the similar results. We found a main effect of the factor work load: [*F*(1,19) = 11.984, *p* = 0.003, η^2^ = 0.387], but no main effect of posture or posture x workload interactions. Subsequent pairwise comparisons revealed a higher accuracy rate in the 1-back (97.12 %) than in the 2-back condition (94.10%). A main effect of the factor posture or interactions between posture and work load could not be found. After integrating sleep quality as a covariate, the ANCOVA revealed a significant three way interaction between posture, workload and PSQI total score [*F*(1,19) = 5.507, *p* = 0.030, η^2^ = 0.225]. The ANCOVA integrating the TICS screening scale yielded no significant main effects or interactions. The ANOVA (examination day x work load) showed significant results for the factor examination day [*F*(1,20) = 7.444, *p* = 0.013, η^2^ = 0.271], work load [*F*(1,20) = 11.656, *p* = 0.003, η^2^ = 0.368] and a day × work load interaction [*F*(1,20) = 25.208, *p* < 0.001, η^2^ = 0.558]. Subsequent pairwise comparisons showed that that participants responded more accurate on the second than on the first day of examination during the 2-back but not the 1-back task (1-back: day 1: 97.18 %, day 2: 97.06 %, *p* = 0.052; 2-back: day 1: 92.86 %, day 2: 95.36, *p* < 0.001).

### PHYSIOLOGICAL DATA

#### Posture × time

**FIGURE 4:**
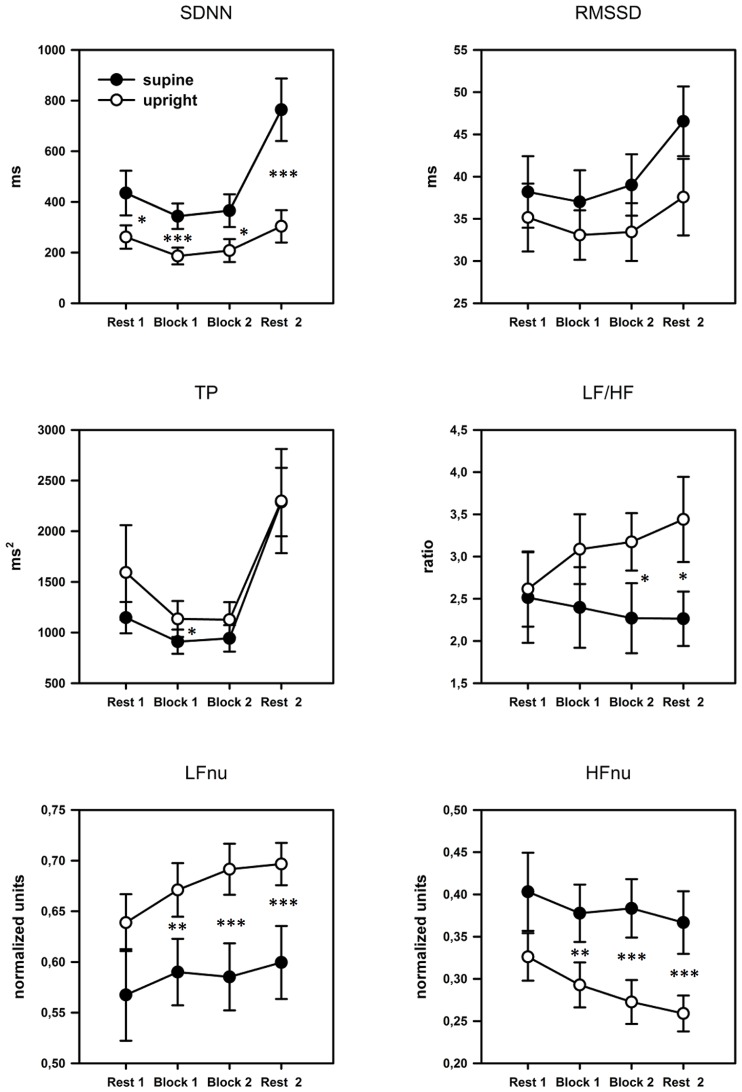
**Profile plots of the time and frequency domain HRV parameters over the four blocks.** Error bars indicate SEM. SDNN, SD of all NN intervals; RMSSD, square root of the mean squared difference between adjacent NN intervals; TP, total power; LF, power in low frequency range; HF, power in high frequency range; nu, normalized units. Pairwise comparisons indicate significant differences between groups: *p < 0.05; **p < 0.01; ***p < 0.001.

As can be seen in **Figure 4**, TP, SDNN, and RMSSD yielded a quadratic time course with attenuated variability during task performance compared to the rest periods before and after the task. The ANOVA revealed significant quadratic main effects of the factor time for all three parameters: SDNN: [*F*(2,40) = 15.939, *p* = 0.001, η^2^ = 0.432], RMSSD: [*F*(2,42) = 8.728, *p* = 0.008, η^2^ = 0.294], TP: [*F*(2,34) = 15.336, *p* = 0.001, η^2^ = 0.422]. Moreover, the ANOVA yielded a significant main effect of the factor posture for SDNN: [*F*(1,21) = 13.414, *p* = 0.001, η^2^ = 0.390], as well as a significant interaction between posture and quadratic time for SDNN [*F*(1,21) = 9.467, *p* = 0.006, η^2^ = 0.311]. For RMSSD and TP no further main effects or interactions could be observed.

Significant main effects of the factor posture could also be found for the normalized parameters LFnu [*F*(1,21) = 15.213, *p* = 0.001, η^2^ = 0.420] and HFnu [*F*(1,21) = 16.290, *p* = 0.001, η^2^ = 0.437] but not for LF/HF. A significant interaction between posture and linear time for the LF/HF ratio [*F*(2,45) = 4.649, *p* = 0.013, η^2^ = 0.181] was also found. Subsequent pairwise comparisons showed that the upright posture was associated with a significant increase in LF/HF: Rest1 < Rest2, *p* = 0.001, and LFnu: Rest1 < Rest2, *p* = 0.001, and a significant decrease in HFnu: Rest1 > Rest2, *p* < 0.001. Please, see **Table 2** for *post hoc* statistics between postures.

**Table 2:** Means and standard errors of postures and time periods (blocks).

	Block	Supine	Upright	*p*
SDNN	1	435.03 (87.88)	261.37 (46.27)	0.025^*^
	2	343.74 (50.25)	186.50 (32.94)	0.001^***^
	3	365.43 (64.78)	208.28 (45.28)	0.026^*^
	4	763.90 (123.41)	303.58 (63.65)	0.001^***^
				
RMSSD	1	38.18 (4.23)	35.15 (4.01)	0.513
	2	37.00 (3.73)	33.07 (2.92)	0.170
	3	39.00 (3.63)	33.43 (3.42)	0.068
	4	46.54 (4.12)	37.56 (4.53)	0.056
				
TP	1	1147.38 (154.49)	1592.29 (467.56)	0.292
	2	910.08 (119.40)	1133.99 (177.47)	0.042^*^
	3	942.51 (130.53)	1126.97 (173.03)	0.197
	4	2288.07 (337.55)	2297.69 (514.33)	0.986
				
LFnu	1	0.56 (0.04)	0.63 (0.02)	0.061
	2	0.59 (0.03)	0.67 (0.02)	0.005^**^
	3	0.58 (0.03)	0.69 (0.02)	0.001^***^
	4	0.59 (0.03)	0.69 (0.02)	0.001^***^
				
HFnu	1	0.40 (0.04)	0.32 (0.02)	0.052
	2	0.37 (0.03)	0.29 (0.02)	0.004^**^
	3	0.38 (0.03)	0.27 (0.02)	0.001^***^
	4	0.36 (0.03)	0.25 (0.02)	0.001^***^
				
LF/HF	1	2.51 (0.53)	2.61 (0.44)	0.835
	2	2.39 (0.47)	3.08 (0.41)	0.133
	3	2.27 (0.41)	3.17 (0.34)	0.029^*^
	4	2.26 (0.32)	3.43 (0.50)	0.013^*^

* *p* < 0.05; **p < 0.01; ***p < 0.001.

#### Posture × workload

We further investigated whether the body posture and workload affected the HRV parameters. The 2 × 2 ANOVA (posture × work load) yielded the following results: significant main effects of the factor posture could be found for: SDNN: [*F*(1,21) = 9.467, *p* = 0.006, η^2^ = 0.311]; LFnu: [*F*(1,21) = 14.750, *p* = 0.001, η^2^ = 0.413] and HFnu: [*F*(1,21) = 15.028, *p* = 0.001, η^2^ = 0.417]. A main effect of work load was found for: RMSSD: [*F*(1,21) = 11.306, *p* = 0.003, η^2^ = 0.350]. TP and LF/HF showed no significant results. No significant interactions could be found for any of the observed parameters. Subsequent pairwise comparisons are presented in **Table 3**.

**Table 3:** Means and standard errors of HRV parameters of posture and work load levels.

	Supine			Upright	
	1-back	2-back	*p*	1-back	2-back	*p*
SDNN	388.19 (70.20)	320.98 (46.07)	0.157	212.63 (35.68)	182.15 (45.58)	0.251
RMSSD	39.56 (3.81)	36.44 (3.60)	0.033^*^	34.66 (3.25)	31.84 (3.16)	0.061
TP	941.61 (126.62)	910.98 (122.38)	0.692	1190.58 (191.36)	1070.38 (164.10)	0.286
LFnu	0.58 (0.03)	0.58 (0.03)	0.820	0.67 (0.02)	0.69 (0.02)	0.254
HFnu	0.38 (0.03)	0.37 (0.03)	0.668	0.29 (0.02)	0.27 (0.02)	0.165
LF/HF	2.41 (0.50)	2.24 (0.40)	0.515	3.05 (0.37)	3.20 (0.38)	0.491

* *p* < 0.05.

## DISCUSSION

Referring back to the hypotheses formulated in the introduction, the main findings of the study are: (1) We could not identify a significant difference in task performance between body postures, neither with respect to RTs nor accuracies. (2) HRV parameters confirmed a higher level of parasympathetic activity in the supine posture and indicated a relative increase of sympathetic activity in the upright position only. (3) Subjects reported a decreasing alertness level during both conditions over time but against our hypothesis without a significant effect of posture. Additionally and most importantly, subjects showed slower RTs the poorer their sleep quality was only in the supine position and not in the upright condition.

In the present study, we observed that general subjective sleep quality as measured by the PSQI correlates with RTs when the N-back task was performed in the supine position. However, no associations could be found when subjects performed the task in the upright position. How can this finding be explained? Subjects performed the task in four blocks of 4 min each over a total duration of 18 min. Although the 1-back and 2-back conditions required a moderate level of cognitive engagement, the fact that the task has to be performed over this extensive length represents a challenging component that required a sustained engagement of working memory and alertness in particular because subjects had to respond to both: N-back cases and non-N-back cases. The maintenance of this cognitive effort over four times 4 min resulted in increased fatigue which was reflected by decreased ratings on the alertness scale of the MDMQ. In order to counteract the diminishing levels of alertness, subjects have to arouse themselves to perform the task adequately. Arousal abilities, however, were markedly impaired during the supine posture as indicated by HRV parameters (please see next paragraph), a finding that has been repeatedly shown in prior studies (Cole, 1989; Caldwell et al., 2000; Caldwell et al., 2003). Nevertheless, performance and RTs did not differ between postures in general but only after considering subjective sleep quality in the analysis. Evidence from prior studies has clearly shown that poor sleep quality can impair the level of alertness (e.g., Miyata et al., 2010). Our study shows that lack of sleep may be compensated by arousal in an upright posture but not in the supine posture and can result in posture-specific slowed central information processing as indicated by slower RTs in the supine posture compared to the sitting posture and increasing RTs with poorer quality of sleep in the supine position only.

However, why does it only affect the RTs and not the accuracy rate? An accuracy feedback was given continuously to subjects that compelled the subjects to avoid errors. Thus subjects strongly focused on accuracy during task performance probably at the expense of RTs, an effect known as “speed accuracy tradeoff” (e.g., Dutilh et al., 2011). Therefore, it is possible that accuracy rate would also be affected if a similar task had been performed without feedback or a harder task with higher error rates.

Regarding the lack of a main effect of postures on RTs and accuracy without including covariates, we may not have had enough power to detect such effects because of confounding learning effects. Though the study protocol included a short training phase of the N-back task, subjects reacted faster on the second day of the experiment. It might be a valuable approach for future studies to investigate the effect of body posture using a task with lower work load or providing extensive training prior to the investigation to minimize such learning effects. A general effect of body posture might also occur if a between-subject design would be used or if the experiment would not be conducted on two consecutive days.

### HRV PARAMETERS

The time domain measures SDNN and RMSSD displayed higher values at the beginning and the end with lower values during task performance. This U-shaped course clearly indicates the mental effort required by the task, which reduces overall HRV compared to the rest periods. The highest values for these two parameters could be observed during the second rest period at the end of the investigation. We can only speculate over this finding. It is possible that it reflects that subjects anticipated the investigation to end and experienced a feeling of relief after the challenging task blocks. However, several studies have reported this increase of sympathetic parameters at the end of fMRI investigations (Chapman et al., 2010; Muehlhan et al., 2011, 2013). Regarding the frequency domain parameters, only the TP showed a quadratic trend also indicating a lower general HRV during task performance. In contrast, the frequency specific parameters LF/HF ratio, LFnu and HFnu showed linear changes with time but only for the upright posture. In particular the ratio between LF/HF increased in the upright posture but remained at the same level during the supine position. This behavior of the LF/HF ratio can be seen as indicative of the flexibility of the autonomic system in the upright posture to execute a dynamic adjustment to current situational circumstances (task performance/increasing tiredness), compensating the impairment of cognitive task performance by poor sleep quality. These adaptational changes in autonomic activity, however, could not be observed when investigations took place in the supine position, which indicates an impairment of autonomic regulation or flexibility. This impairment led to clear differences between postures in parameters associated with sympathetic and parasympathetic activity.

### MOOD DIMENSIONS

We found no evidence for changes in mood dimension in response to the experimental setting itself as it was shown for fMRI experiments (Muehlhan et al., 2011). However, alertness had decreased after the final rest period following task performance compared to the measurement before the task but without significant differences between postures. The last MDMQ was filled out after rest period 2 and not immediately after the task performance which might account for the lack of significant results. It might be possible that alertness decreased stronger in the supine compared to the upright posture during task performance but that the difference disappeared due to relaxation after the final rest period. Prior evidence from an fMRI study suggests that alertness strongly increases during rest periods (Muehlhan et al., 2011) which, however, might not be directly comparable to the present study.

Taken together, the impaired autonomic regulation during the supine position may lead to negative effects of poor sleep quality on cognitive functions. Consequently, it is recommended to control for subjective sleep quality in neuroimaging investigations and to ask the subjects to come well rested to the experiment. Otherwise the comparison of data recorded inside and outside the scanner might be problematic due to different cognitive and physiological conditions. It should be noted that up to one third of the population complain of insomnia and poor sleep quality (Sivertsen et al., 2009; Ohayon, 2011; Schlack et al., 2013). From a clinical viewpoint it should be taken into account that several psychiatric disorders are associated with poor sleep quality (e.g., Matousek et al., 2004; Lijun et al., 2012). The comparison of patients and healthy controls in neuroimaging investigations might produce group effects that do not occur during daily routine which takes place in upright positions. Additionally, such effects may not be related to the disorder but rather to poor sleep quality. The same problem should be considered when different age groups are compared. Regarding the reliability of neuroimaging investigations, our findings add another confounder. Besides anxiety and stress reactions at the beginning of an fMRI experiment (e.g., Lueken et al., 2011, 2012; Muehlhan et al., 2011, 2013) the negative effects of poor sleep quality can increase the inter- and intrasubject variability reducing the available power to detect the targeted effects.

### LIMITATIONS

The findings reported here have to be interpreted considering certain limitations. The results may vary depending on the population studied. Here, we investigated young and healthy participants. It has been shown that working memory performance as well as HRV parameters differ between age groups (e.g., Yeragani et al., 1997; Rajah and D’Esposito, 2005) or patients and healthy controls (e.g., Henseler and Gruber, 2007; Licht et al., 2008). Thus we cannot generalize the findings to such groups. Furthermore, we counterbalanced our study for gender. It is also possible that the effects vary with gender (Perseguini et al., 2011; Young and Leicht, 2011) an effect that we did not consider given our limited sample size. Future studies should investigate the effect of gender.

In the present investigation, we cannot find any effects of chronic stress. It is possible such an effect was missed because the between-subject variance in self-reported chronic stress was not high enough in our “healthy student” sample. Moreover, we used only the screening TICS scale for our analyses. Furthermore, our power to detect differences in RTs was limited by the learning effects mentioned above.

## CONCLUSION

This study demonstrates that poor quality of sleep can reduce cognitive performance in the supine posture without showing an effect in an upright posture. This finding needs particular consideration in neuroimaging investigations undertaking in the supine posture. It is desirable that participants come well rested to the investigation. It would be valuable to screen for poor sleep quality before subjects are invited to the examination or to match participants of different groups in view of their individual sleep quality. It should be considered that sleep quality effects may easily be larger than gender effects, for example**.** Especially study designs that compare patients and healthy controls or different age groups bear the risk of misinterpretations and have to control for sleep quality. With respect to the finding that sleep quality does not affect cognition in the upright posture, neuroimaging investigations in the supine position bears the risk of overestimating the transferability of their results to everyday life activities that take place predominately in upright postures.

## Conflict of Interest Statement

The authors declare that the research was conducted in the absence of any commercial or financial relationships that could be construed as a potential conflict of interest.
